# Association of sleep characteristics with cardiovascular disease risk in adults over 40 years of age: a cross-sectional survey

**DOI:** 10.3389/fcvm.2024.1308592

**Published:** 2024-01-24

**Authors:** Xin-Zheng Hou, Yu-Shan Li, Qian Wu, Qian-Yu Lv, Ying-Tian Yang, Lan-Lan Li, Xue-Jiao Ye, Chen-Yan Yang, Man-Shi Wang, Yan-Fei Lv, Lin-Lin Cao, Shi-Han Wang

**Affiliations:** ^1^ Department of Cardiovascular Diseases, Guang anmen Hospital Affiliated to China Academy of Chinese Medical Sciences, Beijing, China; ^2^ College of Chinese Medicine, Beijing University of Chinese Medicine, Beijing, China; ^3^ Department of Cardiovascular Diseases, Guangwai Hospital, Beijing, China; ^4^ Shanghai Qianhe Technology Co., Ltd., Shanghai, China

**Keywords:** snoring, daytime sleepiness, bedtime, sleep duration, sleep patterns, cardiovascular disease

## Abstract

**Background:**

The relationship between sleep characteristics and cardiovascular disease (CVD) risk has yet to reach a consistent conclusion, and more research needs to be carried out. This study aimed to explore the relationship between snoring, daytime sleepiness, bedtime, sleep duration, and high-risk sleep patterns with CVD risk.

**Methods:**

Data from the National Health and Nutrition Examination Survey (NHANES) 2015–2018 were collected and analyzed. Multivariable logistic regression was used to evaluate the relationship between snoring, daytime sleepiness, bedtime, sleep duration, high-risk sleep patterns, and CVD risk. Stratified analysis and interaction tests were carried out according to hypertension, diabetes and age.

**Results:**

The final analysis contained 6,830 participants, including 1,001 with CVD. Multivariable logistic regression suggested that the relationship between snoring [OR = 7.37,95%CI = (6.06,8.96)], daytime sleepiness [OR = 11.21,95%CI = (9.60,13.08)], sleep duration shorter than 7 h [OR = 9.50,95%CI = (7.65,11.79)] or longer than 8 h [OR = 6.61,95%CI = (5.33,8.19)], bedtime after 0:00 [OR = 13.20,95%CI = (9.78,17.80)] compared to 22:00–22:59, high-risk sleep patterns [OR = 47.73,95%CI = (36.73,62.04)] and CVD risk were statistically significant. Hypertension and diabetes interacted with high-risk sleep patterns, but age did not.

**Conclusions:**

Snoring, daytime sleepiness, excessive or short sleep duration, inappropriate bedtime, and high-risk sleep patterns composed of these factors are associated with the CVD risk. High-risk sleep patterns have a more significant impact on patients with hypertension and diabetes.

## 1 Introduction

Cardiovascular disease (CVD) is the leading cause of morbidity, disability, and death worldwide, including in low-income countries, and has become a significant public health problem (1). In 2023, the American Heart Association reported that the number of CVD patients, including coronary heart disease (CHD), heart failure, hypertension, and stroke, in the U.S. was 127.9 million (2). Early intervention in CVD risk factors can prevent or reduce CVD incidence, which could benefits population health and relieve economic burden (3, 4). Therefore, identifying risk factors for CVD as much as possible is crucial.

About one-third of an individual's life is dedicated to sleep, emphasizing the criticality of quality sleep for overall health and well-being. Short-term sleep deprivation, long-term sleep restriction, circadian rhythm disturbances and untreated sleep disorders have been associated with far-reaching harmful effects on physical health, mental health, and public safety (5). Long-term lack of sleep will increase personal risks and social burdens related to several medical epidemics, including CVD, diabetes, obesity, and cancer (6). Previous studies have also discussed the relationship between sleep characteristics and CVD risk. A large cohort study found that insomnia was an independent risk factor for CVD, particularly in adults without hypertension (7). Another cohort study established a significant correlation between chronic snoring and an increased risk of ischemic heart disease and ischemic stroke, particularly among individuals under 50 years old (8). Other studies have also found that sleep time and daytime sleepiness were related to the increased risk of CVD (9–11).

However, previous studies assessing the relationship between sleep characteristics and CVD risk have yielded inconsistent results. A Dutch cohort study found that shorter sleep duration increased the risk of CVD compared with 7 h of sleep, while longer sleep duration had no such effect (12). Another cohort study involving multi-ethnic people found that both shorter and longer sleep durations were associated with an increased risk of CVD compared with 7 h of sleep duration, and this relationship was not affected by gender (13). In addition, most previous studies only focused on one sleep characteristic, despite various sleep factors being interactive rather than independent. Therefore, evaluating the correlation between multiple sleep characteristics and CVD risk may be more practical and feasible. For example, a study scored five sleep characteristics, such as insomnia and snoring, and found that healthy sleep patterns were related to reduced risk of CVD (14). Therefore, further study on the relationship between sleep and CVD is of great necessity, especially the comprehensive study of multiple sleep characteristics.

Therefore, we used data from the National Health and Nutrition Examination Survey (NHANES) 2015–2018, to assess the correlation between snoring, daytime sleepiness, bedtime, sleep duration, and CVD. This study also evaluated the association between high-risk sleep patterns and CVD risk by combining the above factors.

## 2 Methods

### 2.1 Study design

The data for this study were collected from the NHANES, a cross-sectional survey designed to assess the health and nutritional status of adults and children in the U.S. NHANES collects data every two years using multistage stratified sampling. The survey is unique in that it combines the home interview and the mobile examination. The NHANES interview includes demographic, socioeconomic, dietary, and health-related questionnaires. The examination includes medical, dental, and physiologic measurements and laboratory tests performed by trained medical personnel (15). The National Center for Health Statistics Ethical Review Board approved the study. Informed consent was obtained from all study participants (16). Therefore, no additional informed consent and ethical review were required for our research.

### 2.2 Study population

In this study, information was collected from participants in the 2017–2018 and 2015–2016 survey cycles, and participants were screened according to the following criteria: 1. participants without sleep information were excluded; 2. Participants without cardiovascular disease information were excluded; 3. Participants under the age of 40 were excluded (17). All remaining participants were included in the study.

### 2.3 Independent variable

Participants' sleep characteristics were obtained through questionnaires from trained interviewers at home using a computer-assisted personal interviewing (CAPI) system. The CAPI system was programmed with built-in consistency checks to minimize data entry errors. This study collected information on snoring, daytime sleepiness, bedtime, and sleep duration. Snoring was determined by asking participants, “ How often did you snore while you were sleeping?” and participants answered “ Never” or “Rarely, 1–2 nights a week” were defined as “no”, while responses of “Occasionally, 3–4 nights a week” or “Frequently, 5 nights a week and more” were defined as “yes”. Daytime sleepiness was determined by asking participants, “ How often did you feel excessively or overly sleepy during the day?” and participants answered “ Never” or “Rarely, 1–2 nights a week” were defined as “no”, while responses of “Occasionally, 3–4 nights a week” or “Frequently, 5 nights a week and more” were defined as “yes”. Bedtime was obtained by asking participants, “ What time do you usually sleep on weekdays or workdays?”. We categorized bedtimes into five categories using 21:00, 22:00, 23:00, and 00:00 as cutoff points. After asking participants about their weekday awakening time, the interval between awakening time and bedtime was sleep duration, which we categorized into three classifications using 7 and 8 h as cutoff points. The division of rest and sleep duration was determined by previous studies (18, 19). In addition, we defined sleep patterns by combining the four sleep characteristics mentioned above and classified sleep patterns as medium-low-risk and high-risk. The simultaneous occurrence of snoring, daytime drowsiness, sleeping for less than 7 or more than 8 h, and not going to bed between 22:00 and 23:00 was considered high-risk sleep patterns; otherwise, it was regarded as the medium-low-risk sleep pattern.

### 2.4 Dependent variable

CVD information was obtained through interviews between interviewers and participants. CVD in this study refers to CHD and stroke. CHD information was obtained by asking participants: “Has a doctor or other health professional ever told you that you had coronary heart disease?” “ Has a doctor or other health professional ever told you that you had angina, also called angina pectoris?” or “Has a doctor or other health professional ever told you that you had a heart attack (also called myocardial infarction)?” If they answered “Yes” to any of the above questions, they were considered CHD. Stroke was defined by asking the participant, “ Has a doctor or other health professional ever told you that you had a stroke?” if the participant answered “yes”, they were considered to have a stroke. Participants with CHD or stroke were defined to have CVD.

### 2.5 Covariates

Covariates included three components: demographics, history, and laboratory tests. Demographic information included gender, age, race, marriage, education, and family poverty income ratio (PIR). Medical history included hypertension, diabetes, cancer, smoking and alcohol use, and body mass index (BMI). Laboratory tests collected data on triglyceride (TG) and low density lipoprotein cholesterol (LDL-C). The trained interviewers used the CAPI system to conduct demographic and history questionnaires at the respondents' homes to obtain corresponding information. This study reclassified some of the original data. Marital status was divided into two categories according to whether the participant had a sexual partner. Participants who were married and cohabiting were defined as “yes”, and participants who were widowed, divorced, separated, and unmarried was described as “no”. Education level was divided into two categories, with high school as the boundary. PIR is divided into three equal parts. History of hypertension, diabetes, and cancer was obtained by asking participants the following questions: “Have you ever been told by a doctor or other health professional that you had hypertension, also called high blood pressure?” “Have you ever been told by a doctor or health professional that you had diabetes or sugar diabetes?” and “Have you ever been told by a doctor or other health professional that you had cancer or a malignancy of any kind?”. Smoking and drinking history were determined by asking participants, “Have you smoked at least 100 cigarettes in your entire life?” or “Was there ever a time or times in your life when you drank 4(female)/5(male) or more drinks of any kind of alcoholic beverage almost every day?”. Participants in the above questions answered “yes” or “no”, and refusing to answer was regarded as missing relevant data. After measuring the participants’ standing height and weight, BMI was calculated using the formula weight (Kg)/height (m) *^2^. Serum samples for laboratory examination were processed, stored, and shipped to the University of Minnesota for analysis. LDL-C and TG were obtained by standard biochemical profiling using a Beckman Synchron LX20.

### 2.6 Statistic analysis

Participants were divided into two groups according to whether they had CVD or not to describe the characteristics of the study population. Continuous variables were expressed as mean and standard deviation or median and quartile, and the *t*-test or Kruskal–Wallis rank sum test was selected for hypothesis testing according to applicable conditions. Classified variables were expressed as absolute numbers and percentages, and the chi-square test was used for hypothesis testing.

Multivariable logistics regression evaluated the relationship between snoring, daytime sleepiness, bedtime, sleep duration and CVD risk respectively. Bedtime and sleep duration were set as dummy variables when included in the model as multi-classification variables. Sleep patterns based on the four sleep characteristics above were also evaluated with the risk of CVD. In addition, CHD and stroke were separately analyzed as dependent variables.

To ensure the robustness of the results, we constructed several regression models. In Model 1, we only adjusted for demographic variables, including age, gender, race, marriage, education level, and PIR. Age was converted into a binary variable and included in the model. In Model 2, we further adjusted for smoking, drinking, and BMI in addition to variables in Model 1. BMI was converted into categorical variables using 18.5 and 25 as cut-off points and then included in the model. We additionally included the history of hypertension, diabetes, and cancer in Model 3 in addition to variables adjusted in Model 2. TG and LDL-C were converted into three-category variables and included in Model 3.

To further explore whether the influence of high-risk sleep patterns on CVD risk was consistent among different populations, we also conducted stratified analysis according to hypertension, diabetes, and age. To verify the interaction, we added the multiplication term of sleep patterns and the above factors to model 3.

Statistics and theory drove the screening of covariates While we incorporated variables with statistical significance in univariate analysis into the regression model, we also included some covariates that may theoretically affect the outcome variables, to maximize the control of confounding bias. The sample size should meet the principle of 10 events per variable (EPV), which means that the group with less sample size of dependent variables was at least ten times the number of variables in the model.

To avoid the selection bias that the lack of covariate data may cause, we treated the missing variables as follows: for classified variables, the missing values were recorded as the “missing” group and included in the analysis; for continuous variables, after being converted into classified variables, the missing values were recorded as the “missing” group and then included in the study.

Data analysis was completed using the IBM SPSS Statistics software version 26.0 (IBM Corp., Armonk, N.Y., USA). *P* < 0.05 on both sides was considered statistically significant. Depending on the rules for selecting weight values offered on the NHANES website, “Full Sample Two-Year Mobile Examination Center Exam Weight” was Selected as representative weighting value (20).

## 3 Results

**Figure 1:**
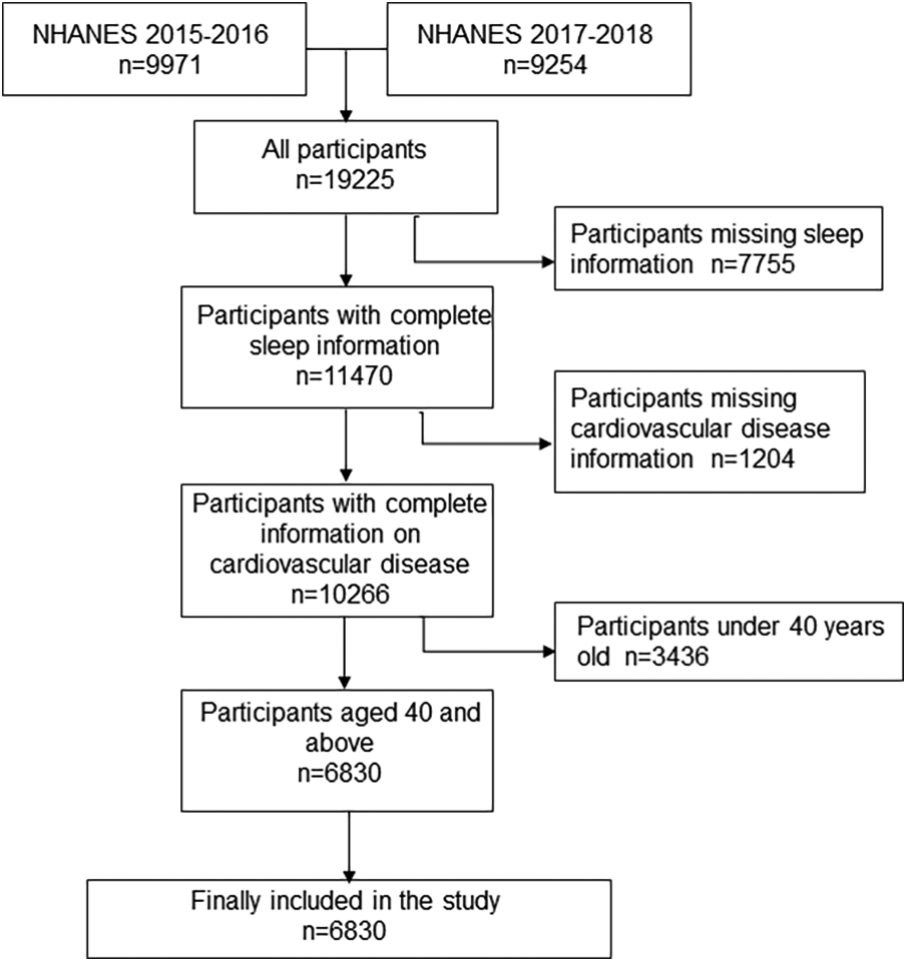
Study population screening flow chart.

The study population screening process is shown in Figure 1. A total of 19,225 participants took part in the NHANES 2015–2018. After initial screening according to inclusion and exclusion criteria, 6,830 participants, with complete information on independent and dependent variables, were included in the study.

The participants were divided into two groups based on whether they had cardiovascular disease. One thousand and one participants had cardiovascular disease. They had higher likelihoods of snoring, daytime sleepiness, short or long sleep durations, and going to bed after midnight, constituting a high proportion of their high-risk sleep patterns. Univariate analysis showed that race, marital status, education level, PIR, smoking, alcohol use, BMI, hypertension, and diabetes might affect cardiovascular disease, so they were included in the multiple regression model. Additionally, age, sex, LDL-C, TG, and cancer were also included in the multiple regression model because, according to theoretical knowledge they might also be associated with cardiovascular disease. The detailed characteristics of the study population are shown in Table 1.

**Table 1:** Study population characteristics.

Variables	Total (*n* = 6,830)	Participants without CVD (*n* = 5,829)	Participants with CVD (*n* = 1,001)	*P*-value
Demographic characteristics				
Age(year),Mean ± SD	59.97 ± 12.00	59.97 ± 12.01	59.96 ± 11.93	0.99
Gender, *n* (%)				0.77
Male	3,355 (49.12)	2,859 (49.05)	496 (49.55)	
Female	3,475 (50.88)	2,970 (50.95)	505 (50.45)	
Race, *n* (%)				<0.01
Mexican American	970 (14.2)	811 (13.91)	159 (15.88)	
Other HISPANIC	803 (11.76)	662 (11.36)	141 (14.09)	
Non-hispanic white	2,394 (35.05)	2,184 (37.47)	210 (20.98)	
Non-hispanic black	1,510 (22.11)	1,303 (22.35)	207 (20.68)	
Other race	1,153 (16.88)	869 (14.91)	284 (28.37)	
Education level, *n* (%)				<0.01
High school below	1,621 (23.73)	1,301 (22.32)	320 (31.97)	
High school and above	5,201 (76.15)	4,523 (77.59)	678 (67.73)	
Missing	8 (0.12)	5 (0.09)	3 (0.30)	
Marriage, *n* (%)				<0.01
Yes	4,408 (64.54)	3,748 (64.30)	660 (65.93)	
No	2,417 (35.39)	2,080 (35.68)	337 (33.67)	
Missing	5 (0.07)	1 (0.02)	4 (0.40)	
PIR, *n* (%)				<0.01
T1	1,981 (29)	1,670 (28.65)	311 (31.07)	
T2	1,994 (29.19)	1,699 (29.15)	295 (29.47)	
T3	2,003 (29.33)	1,751 (30.04)	252 (25.17)	
Missing	852 (12.47)	709 (12.16)	143 (14.29)	
BMI(Kg/m*^2^), Mean ± SD	29.96 ± 6.92	30.37 ± 7.01	27.57 ± 5.76	<0.01
Laboratory examination				
LDL-C(mmol/L), Mean ± SD	2.93 ± 0.97	2.93 ± 0.97	2.95 ± 0.96	0.67
TG(mmol/l), M (Q₁, Q₃)	1.12 (0.78–1.61)	1.12 (0.78–1.63)	1.15 (0.79–1.59)	0.95
Medical history				
Drinking, *n* (%)				<0.01
Yes	927 (13.57)	819 (14.05)	108 (10.79)	
No	4,247 (62.18)	3,683 (63.18)	564 (56.34)	
Missing	1,656 (24.25)	1,327 (22.77)	329 (32.87)	
Smoking, *n* (%)				<0.01
Yes	3,052 (44.69)	2,684 (46.05)	368 (36.76)	
No	3,774 (55.26)	3,142 (53.90)	632 (63.14)	
Missing	4 (0.06)	3 (0.05)	1 (0.10)	
Hypertension, *n* (%)				<0.01
Yes	3,291 (48.18)	2,903 (49.80)	388 (38.76)	
No	3,532 (51.71)	2,921 (50.11)	611 (61.04)	
Missing	7 (0.1)	5 (0.09)	2 (0.20)	
Diabetes, *n* (%)				<0.01
Yes	1,435 (21.01)	1,273 (21.84)	162 (16.18)	
No	5,391 (78.93)	4,552 (78.09)	839 (83.82)	
Missing	4 (0.06)	4 (0.07)	0 (0.00)	
Cancer, *n* (%)				0.17
Yes	939 (13.75)	820 (14.07)	119 (11.89)	
No	5,887 (86.19)	5,005 (85.86)	882 (88.11)	
Missing	4 (0.06)	4 (0.07)	0 (0.00)	
Sleep characteristics				
Snore, *n* (%)				<0.01
No	3,196 (46.79)	3,069 (52.65)	127 (12.69)	
Yes	3,634 (53.21)	2,760 (47.35)	874 (87.31)	
Daytime sleepiness, *n* (%)				<0.01
No	5,202 (76.16)	4,885 (83.81)	317 (31.67)	
Yes	1,628 (23.84)	944 (16.19)	684 (68.33)	
Sleep duration, *n* (%)				<0.01
7–8 h	3,093 (45.29)	2,975 (51.04)	118 (11.79)	
Less than 7 h	1,630 (23.87)	1,184 (20.31)	446 (44.56)	
More than 8 h	2,107 (30.85)	1,670 (28.65)	437 (43.66)	
Bedtime, *n* (%)				<0.01
Before 21:00	446 (6.53)	364 (6.24)	82 (8.19)	
21:00–21:59	1,050 (15.37)	864 (14.82)	186 (18.58)	
22:00–22:59	2,044 (29.93)	1,992 (34.17)	52 (5.19)	
23:00–23:59	1,731 (25.34)	1,462 (25.08)	269 (26.87)	
After 24:00	1,559 (22.83)	1,147 (19.68)	412 (41.16)	
Sleep patterns. *N* (%)				<0.01
Medium-low-risk	6,361 (93.13)	5,747 (98.59)	614 (61.34)	
High-risk	469 (6.87)	82 (1.41)	387 (38.66)	

CVD, cardiovascular disease; PIR, Family income-poverty ratio (T1<=1.32,1.32 < T2<=2.78, 2.78 < T3); BMI, body mass index; LDL-C, low density lipoprotein-cholesterol; TG, triglyceride.

The relationship between different sleep characteristics and CVD risk is shown in Table 2. Although we have constructed several regression models to examine the robustness of the results, their results were similar. After fully adjusting various potential confounding factors, snoring [*OR = 7.37,95%CI = (6.06,8.96)*] and daytime sleepiness [*OR = 11.21,95%CI = (9.60,13.08)*] were positively correlated with CVD. Compared with the participants who went to bed from 22: 00 to 22: 59, those who went to bed at other periods also had a higher CVD risk. The effect values were before 21:00 [*OR = 8.29,95%CI = (5.73,11.99)*], 21:00–21:59 [*OR = 7.95,95%CI = (5.77,10.95)*], 23:00–23:59 [*OR = 6.78,95%CI = (4.99,9.20)*], after 0:00 [*OR = 13.20,95%CI = (9.78,17.80)*] respectively. Sleep duration shorter [*OR = 9.50,95%CI = (7.65,11.79)*], or longer [*OR = 6.61,95%CI = (5.33,8.19)*], than 7–8 h was positively associated with CVD risk. The high-risk sleep patterns [*OR = 47.73,95%CI = (36.73,62.04)*] formed by the superposition of the above dangerous sleep characteristics greatly increased the CVD risk. CHD and stroke also showed similar results when analyzed separately as dependent variables. Negative sleep characteristics have a greater influence on CHD than stroke, as shown in Tables 3, 4.

**Table 2:** Relationship between sleep characteristics and CVD risk.

	Model 1	Model 2	Model 3
Snore			
No	Reference	Reference	Reference
Yes	7.62 (6.28,9.26)	7.55 (6.21,9.17)	7.37 (6.06,8.96)
Daytime sleepiness			
No	Reference	Reference	Reference
Yes	11.29 (9.70,13.15)	11.34 (9.72,13.22)	11.21 (9.60,13.08)
Sleep duration			
7–8 h	Reference	Reference	Reference
Less than 7 h	9.63 (7.77,11.94)	9.56 (7.71,11.86)	9.50 (7.65,11.79)
More than 8 h	6.69 (5.41,8.28)	6.71 (5.42,8.31)	6.61 (5.33,8.19)
Bedtime			
Before 21:00	8.46 (5.86,12.20)	8.49 (5.88,12.26)	8.29 (5.73,11.99)
21:00–21:59	8.31 (6.04,11.43)	8.20 (5.95,11.28)	7.95 (5.77,10.95)
22:00–22:59	Reference	Reference	Reference
23:00–23:59	6.96 (5.13,9.44)	6.90 (5.08,9.37)	6.78 (4.99,9.20)
After 0:00	13.70 (10.17,18.46)	13.49 (10.01,18.19)	13.20 (9.78,17.80)
Sleep patterns			
Medium-low-risk	Reference	Reference	Reference
High-risk	43.14 (33.45,55.64)	44.90 (34.72,58.07)	47.73 (36.73,62.04)

Results are expressed as odds ratio (OR) and 95% confidence interval (CI).

The following variables were adjusted:

Model 1: age, gender, race, marriage, education level, PIR.

Model 2: age, gender, race, marriage, education level, PIR, smoking, drinking, BMI.

Model 3: age, gender, race, marriage, education level, PIR, smoking, drinking, BMI, hypertension, diabetes, cancer, TG, LDL-C.

**Table 3:** Relationship between sleep characteristics and CHD risk.

	Model 1	Model 2	Model 3
Snore			
No	Reference	Reference	Reference
Yes	8.77 (6.91,11.12)	8.66 (6.82,10.99)	8.51 (6.70,10.80)
Daytime sleepiness			
No	Reference	Reference	Reference
Yes	5.59 (4.76,6.56)	5.54 (4.72,6.51)	5.45 (4.64,6.41)
Sleep duration			
7–8 h	Reference	Reference	Reference
Less than 7 h	21.120 (15.00,29.74)	20.94 (14.87,29.51)	20.79 (14.75,29.30)
More than 8 h	17.54 (12.49,24.63)	17.60 (12.53,24.73)	17.38 (12.37,24.43)
Bedtime			
Before 21:00	39.91 (19.73,80.74)	40.03 (19.78,81.02)	39.36 (19.44,79.69)
21:00–21:59	36.97 (18.77,72.85)	36.54 (18.54,72.02)	35.79 (18.16,70.55)
22:00–22:59	Reference	Reference	Reference
23:00–23:59	30.15 (15.41,59.00)	29.91 (15.29,58.54)	29.58 (15.12,57.90)
After 0:00	57.82 (29.69,112.61)	57.00 (29.26,111.03)	56.05 (28.77,109.22)
Sleep patterns			
Medium-low-risk	Reference	Reference	Reference
High-risk	24.68 (19.87,30.67)	25.28 (20.30,31.49)	25.63 (20.54,31.97)

Results are expressed as odds ratio (OR) and 95% confidence interval (CI).

The following variables were adjusted:

Model 1: age, gender, race, marriage, education level, PIR.

Model 2: age, gender, race, marriage, education level, PIR, smoking, drinking, BMI.

Model 3: age, gender, race, marriage, education level, PIR, smoking, drinking, BMI, hypertension, diabetes, cancer, TG, LDL-C.

**Table 4:** Relationship between sleep characteristics and stroke risk.

	Model 1	Model 2	Model 3
Snore			
No	Reference	Reference	Reference
Yes	3.13 (2.46,3.99)	3.07 (2.41,3.91)	2.95 (2.32,3.77)
Sleep duration			
7–8 h	Reference	Reference	Reference
Less than 7 h	2.86 (2.23,3.67)	2.79 (2.18,3.59)	2.75 (2.14,3.53)
More than 8 h	1.54 (1.18,2.00)	1.52 (1.17,1.98)	1.48 (1.13,1.93)
Bedtime			
Before 21:00	2.31 (1.43,3.76)	2.31 (1.42,3.75)	2.22 (1.36,3.61)
21:00–21:59	2.58 (1.78,3.75)	2.52 (1.74,3.66)	2.41 (1.66,3.50)
22:00–22:59	Reference	Reference	Reference
23:00–23:59	2.38 (1.69,3.35)	2.34 (1.66,3.29)	2.26 (1.60,3.18)
After 0:00	3.86 (2.78,5.35)	3.76 (2.72,5.22)	3.64 (2.62,5.04)
Sleep patterns			
Medium-low-risk	Reference	Reference	Reference
High-risk	9.63 (7.58,12.24)	9.60 (7.54,12.22)	9.65 (7.56,12.31)

Results are expressed as odds ratio(OR) and 95% confidence interval (CI).

The following variables were adjusted:.

Model 1: age, gender, race, marriage, education level, PIR.

Model 2: age, gender, race, marriage, education level, PIR, smoking, drinking, BMI.

Model 3: age, gender, race, marriage, education level, PIR, smoking, drinking, BMI, hypertension, diabetes, cancer, TG, LDL-C.

The results of stratified analysis showed that high-risk sleep patterns and increased risk of CVD were population-specific. The CVD risk of participants with hypertension [*OR = 74.55,95%CI = (49.84,111.50)*] was more affected by high-risk sleep patterns than those without hypertension [*OR = 31.96,95%CI = (22.72,44.96)*]. Similarly, the CVD risk of diabetic participants was more easily affected by high-risk sleep patterns [*OR = 99.01,95%CI = (52.94,185.15)* vs. *OR = 40.11,95%CI = (30.04,53.56)*]. Participants over 60 years old were also more susceptible to the impact of high-risk sleep patterns on CVD risk [*OR = 59.33,95%CI = (40.90,86.06)* vs. *OR = 37.84,95%CI = (26.04,54.98)*]. Still, interaction analysis between age and sleep patterns was not statistically significant (*P = 0.08*), which was worth further exploration. The above results are shown in Table 5.

**Table 5:** Relationship between high-risk sleep patterns and CVD risk in different populations.

	Model 1	Model 2	Model 3	*P* for interaction
Hypertension				<0.01
Yes	68.25 (46.14,100.96)	71.87 (48.28,106.98)	74.55 (49.84,111.50)	
No	30.63 (21.85,42.92)	31.36 (22.33,44.05)	31.96 (22.72,44.96)	
Diabetes				<0.01
Yes	90.03 (49.09,165.10)	93.27 (50.60,171.91)	99.01 (52.94,185.15)	
No	37.43 (28.20,49.67)	38.76 (29.11,51.60)	40.11 (30.04,53.56)	
Age				0.08
<60	33.98 (23.69,48.76)	36.38 (25.19,52.55)	37.84 (26.04,54.98)	
≥60	54.73 (38.06,78.71)	55.28 (38.37,79.64)	59.33(40.90,86.06)	

Results are expressed as odds ratio (OR) and 95% confidence interval (CI).

The following variables were adjusted:

Model 1: age, gender, race, marriage, education level, PIR.

Model 2: age, gender, race, marriage, education level, PIR, smoking, drinking, BMI.

Model 3: age, gender, race, marriage, education level, PIR, smoking, drinking, BMI, hypertension, diabetes, cancer, TG, LDL-C. When a variable was used as a hierarchical standard, it was not included in the model.

## 4 Discussion

This study found that snoring, daytime sleepiness, a sleep duration of less than 7 h or more than 8 h, as well as going to bed outside the 22:00–23:00 time frame, were associated with a higher risk of CVD. The high-risk sleep pattern, characterized by the above sleep factors, significantly increased the risk of CVD. Stratified analysis showed that high-risk sleep patterns significantly impacted CVD risk among people with hypertension and diabetes. No interaction between high-risk sleep patterns and age was found.

Univariate analysis found that participants with CVD had lower educational levels and higher poverty rates. However, unlike previous findings, this study's results showed that CVD participants had lower BMI, smoking and drinking proportion, which may be because participants with CVD have received good health education and given up these unhealthy living habits. Still, the cross-sectional survey research design cannot demonstrate this causal relationship.

The mechanisms of the association between sleep and CVD remain unknown. Sleep is an essential part of biological rhythm. Healthy sleep requires proper bedtime, enough sleep time and high-quality sleep without abnormal behaviors such as snoring and nightmares, which is essential for cognitive function, mental health, physical health, and mood (21). Some unhealthy sleep characteristics can harm health in multiple ways. When habitual snoring occurs during sleep, the upper airway is narrowed or blocked, resulting in hypoxia. Chronic hypoxia can lead to inflammatory reactions, oxidative stress and chronic sympathetic nerve activation, which can induce or aggravate atherosclerosis. Moreover, snoring-related energy may cause atherosclerotic plaque rupture (22–25). Shortening sleep duration is related to endocrine disorders and circadian rhythm disorders, which may increase the CVD risk (26). For example, shortening sleep time will decrease testosterone, melatonin and leptin secretion and increase auxin levels, which are risk factors for CVD (27–31). The above mechanisms may synergistically increase CVD risk (14).

Our findings were consistent with findings from previous studies. Two meta-analyses found that longer sleep time was related to an increased risk of CHD and stroke (32, 33). A large cohort study found that insomnia can increase the incidence of acute myocardial infarction (34). Insomnia and stroke also showed a similar relationship (35). Daytime sleepiness was associated with new strokes and cardiovascular events, and this relationship was more evident in older people (36). Habitual snoring and obstructive sleep apnea were risk factors for stroke and CHD (37). The above research only focuses on one sleep characteristic, but various sleep risk factors are not independent. For example, people who habitually snore are more likely to be sleepy during the day and have longer sleep durations (38, 39). Therefore, studying the superposition of various sleep characteristics is more practical. A cohort study in China found that there was a dose-response relationship between sleep health score and CVD risk after adding snoring, daytime sleepiness, insomnia, and sleep duration. This was consistent with our research conclusion, that was, the superposition of various dangerous sleep factors would increase the CVD risk (40). Our study further carried out hierarchical analysis and found that this relationship was more significant among participants with hypertension and diabetes. A survey among people with diabetes also confirmed our conclusion (19).

Although sleep health is getting more and more attention, more attention should be paid to clinical and public health efforts. Many resources are invested in promoting healthy nutrition, regular exercise, and reducing risky behaviors like smoking. In contrast, projects to promote healthy sleep still need to be developed (5). Sleep is a multidisciplinary field, and cognitive behavioral intervention is the first choice to treat sleep problems. However, only 6% of clinical psychology programs offer formal teaching courses in sleep medicine (41). Another study found that 95% of the respondents said they had yet to receive clinical sleep training during their postgraduate years and internship years (42).

Our study found that adverse sleep characteristics, such as snoring, daytime sleepiness, excessive or insufficient sleep duration, and inappropriate bedtime, were CVD risks. Furthermore, the accumulation of these adverse sleep characteristics can significantly increase CVD risk. Our research results indicate that in addition to traditional CVD risk factors, above sleep characteristics may also contribute to the development of CVD. Therefore, it is important to consider incorporating sleep monitoring and sleep therapy into CVD prevention and treatment strategies. Previous small-scale clinical studies have found that patients with obstructive sleep apnea syndrome (OSAS) who receive continuous positive airway pressure (CPAP) treatment can improve insulin resistance, oxidative stress, and inflammatory responses, thereby reducing CVD risk (43). However, other studies have also found that treating OSAS does not improve endothelial function (43). Whether improving sleep can reduce CVD risk remains controversial, which points to the need for further research in this area.

The strength of this study is to construct multiple regression models with different independent and dependent variables and fully adjust the potential confounding factors to describe the relationship between other sleep characteristics and CVD risk to the maximum extent. Secondly, we evaluated the relationship between individual sleep characteristics, high-risk sleep patterns formed by the superposition of various dangerous sleep characteristics and CVD risk, and carried out a hierarchical analysis to identify population specificity, which made the research more practical. In addition, the data of this study comes from NHANES, of which the research design is rigorous, and the data is reliable. As for limitations, this study, as a cross-sectional survey, cannot make causal inferences, therefore the results and conclusions of this study cannot clearly explain the causal relationship between various sleep characteristics and CVD risk. In addition, due to the limitation of sample size, sleep patterns are only divided into high-risk and medium-low-risk groups. High-risk sleep patterns contain various dangerous characteristics, which may lead to a relatively large effect value. Subsequent research can further refine sleep patterns by expanding the sample size. Finally, the data on sleep from NHANES comes from a questionnaire survey. During the survey, CAPI system was programmed with limited built-in consistency checks to reduce data entry errors. Other measures are taken to ensure the integrity, consistency, and analytical usefulness of the data. These measures can control system errors (44). Numerous studies have been published based on the sleep survey. However, there may still be information bias compared to objective data obtained through sleep breathing detection.

## Data Availability

The raw data supporting the conclusions of this article will be made available by the authors, without undue reservation.
